# Association of Health Record Visualizations With Physicians’ Cognitive Load When Prioritizing Hospitalized Patients

**DOI:** 10.1001/jamanetworkopen.2019.19301

**Published:** 2020-01-15

**Authors:** Ari H. Pollack, Wanda Pratt

**Affiliations:** 1 Division of Nephrology, Seattle Children’s Hospital, Seattle, Washington; 2 Department of Pediatrics, University of Washington School of Medicine, Seattle; 3 Information School, University of Washington, Seattle

## Abstract

**Importance:**

Current electronic health records (EHRs) contribute to increased physician cognitive workload when completing clinical tasks.

**Objective:**

To assess the association of different design features of an EHR-based information visualization tool with the cognitive load of physicians during the clinical prioritization process.

**Design, Setting, and Participants:**

This cross-sectional study included a convenience sample of 29 attending physicians at Seattle Children’s Hospital, a large tertiary academic pediatric hospital. Data collection took place from August 2017 through October 2017, and analysis occurred from August to October 2018.

**Exposure:**

Physician participants used 3 prototypes with novel visualizations of simulated EHR data that highlighted 1 of 3 key patient characteristics, as follows: (1) acuity, (2) clinical problem list, and (3) clinical change.

**Main Outcomes and Measures:**

Cognitive workload was measured using the NASA Task Load Index (TLX) scale (range, 1-100, with lower scores indicating lower cognitive workload). Cognitive workload was assessed for the 2 following clinical prioritization tasks: (1) finding information for a specific patient and (2) comparing results among patients for each prototype. Participants ranked 5 hypothetical patients from having the highest to the lowest priority in each design.

**Results:**

A total of 29 physician participants (15 [52%] men; 14 [48%] women; mean [range] age, 43 [35-58] years; mean [range] time in practice, 11 [3-30] years) completed the study. For task 1, the prototype highlighting clinical change was associated with lower median (interquartile range) NASA TLX scores compared with the prototype highlighting acuity (30.3 [15.2-41.6] vs 48.5 [18.7-59.3]; *P* = .02). For task 2, the prototype highlighting clinical change was associated with lower median (interquartile range) NASA TLX scores compared with the prototype highlighting the clinical problem list (29.1 [16.3-50.8] vs 43.5 [26.6-55.9]; *P* = .02). The prototype highlighting clinical change had the lowest TLX score in 17 of 29 rankings (59%) for task 1 (χ^2^_4_ = 24.4; *P* < .001) and 18 of 29 rankings (62%) for task 2 (χ^2^_4_ = 17.2; *P* = .002).

**Conclusions and Relevance:**

In this study, well-designed EHR-based information visualizations that highlighted and featured clinically meaningful information patterns significantly reduced physician cognitive workload when prioritizing patient needs.

## Key Points

**Question:**

Can information visualization tools within electronic health records reduce the cognitive workload for physicians when identifying which patients have the highest-priority care needs?

**Findings:**

In this cross-sectional study of 29 physicians, information visualization tools that identified and highlighted clinically meaningful patterns were associated with a significantly lower cognitive workload compared with tools that required physicians to spend more time searching for similar information.

**Meaning:**

Electronic health records that use well-designed information visualization tools have the potential to reduce cognitive workload among physicians.

## Teaser

This cross-sectional study assesses the association of different design features of an electronic health record–based information visualization tool with the cognitive load of physicians during the clinical prioritization process.

## Introduction

Hospitalized patients generate hundreds to thousands of new pieces of clinical data each day, requiring physicians to review, process, prioritize, and ultimately take action on tens of thousands of different data points when managing multiple patients.^1^ Identifying which patients to prioritize requires physicians to search and filter this large volume of information to find the clinically meaningful and important details and distinguish between patients with high-priority and low-priority care needs. Without the proper support, this overwhelming task leads to information overload and increases physician cognitive workload, ie, the effort required to identify, use, and maintain data in working memory.^2^ Despite their initial promise, electronic health records (EHRs) have failed to help physicians reduce their cognitive workload. Owing to a lack of intelligent or effective EHR information visualization support tools, physicians receive little support for recognizing clinically relevant patterns and recreating the patient’s story.^3,4,5,6^ These deficiencies cause physicians to spend a significant amount of time searching for and synthesizing results across multiple EHR sections, thus increasing their cognitive workload as they interpret results and make clinical decisions.^7,8^

Systems that aggregate and visualize data to highlight clinically meaningful patterns are needed to reduce the cognitive workload associated with EHR use. In this study, we identified EHR visualization strategies associated with decreases in the cognitive workload experienced by physicians when identifying which patients to see first (ie, the clinical prioritization process), ensuring that the most urgent needs are addressed first. In previous work,^9^ we identified the key data elements used during the clinical prioritization process, with physicians synthesizing details of their patients’ acuity, clinical problem list, and, most importantly, changes in clinical status. In this study, we assessed the cognitive workload associated with the use of 3 novel visualizations of EHR data designed to support physicians during the clinical prioritization process by exploring the association of different data organization and visualization strategies with cognitive workload. We hypothesized that well-designed visualization tools would be associated with reductions in cognitive workload and could support physicians during the clinical prioritization process.

## Methods

This cross-sectional study was approved by the Seattle Children’s Hospital institutional review board and was conducted at Seattle Children’s Hospital, a tertiary care pediatric hospital in Seattle, Washington. Participants were attending-level physicians at Seattle Children’s Hospital and were invited to participate via a direct email request from 1 of us (A.H.P.). Potential participants were purposefully selected by 1 of us (A.H.P.) to maximize diversity based on medical specialty, sex, age, and clinical experience. Written informed consent was obtained from all participants. We followed the Strengthening the Reporting of Observational Studies in Epidemiology (STROBE) reporting guideline for cross-sectional studies.^10^

### Visualization Development

Before recruitment, the research team developed 3 high-fidelity prototypes that used novel visualizations of simulated EHR data. These prototypes were designed to support a key step of the clinical prioritization process, ie, helping clinicians answer the question, “Which patient should I see next?” Each prototype used a different visualization strategy to present clinical data to participants. The final designs were informed by 2 previous studies, as follows: focus groups with practicing clinicians that identified information needs during the clinical prioritization process^9^ and a human-centered participatory design session during which clinicians had an opportunity to design a clinical prioritization support tool (unpublished data).^11^ Each prototype highlighted and organized data around 1 of 3 key patient characteristics (ie, acuity, clinical problem list, or clinical change) identified in our previous work.^9^ Clinical data included details and results (ie, laboratory tests, medications, vital sign measurements, and clinical notes) on 5 fictional hospitalized patients during a 24-hour period. To accurately assess the cognitive workload associated with the use of each prototype, the data in each prototype had to be functionally equivalent yet clinically different. Therefore, each prototype contained the same amount of clinical information, including the number of laboratory test results, medications, vital sign measurements, and clinical notes. In addition, each prototype had the same number of abnormal results as well as scheduled and as-needed medications. However, the specific details (eg, specific laboratory test results or medications) changed in each prototype, so our participants would not remember details between prototypes. Thus, any differences associated with the complexity of using a particular prototype were associated with its specific design features and not with similarities or differences in the clinical content. To accomplish this task, we developed and used a 5-step process to generate synthetic patient data explicitly for the purpose of evaluating novel health information technology, which has been previously reported.^12^

Each prototype first provided users with a summary of the clinical status of the 5 hypothetical patients via a dashboard-style overview to support quick comparisons among patients. Selecting an individual patient opened a more detailed view that provided insight into the summary view visualization. The fully interactive prototypes consisted of multiple pages of clinical information linked through design-specific features unique to each prototype.

#### Prototype 1: Acuity View

**Figure 1.**
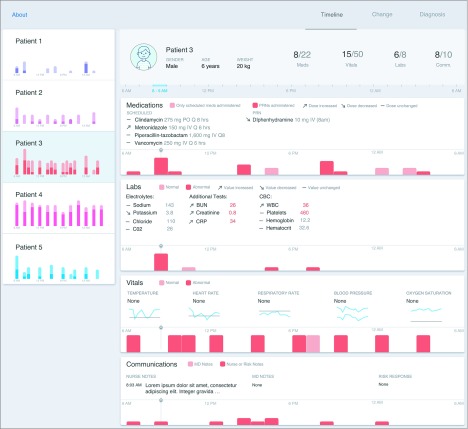
Acuity Prototype The acuity prototype presented an overview of all 5 hypothetical patients on the left, displaying activity levels as a vertical bar, with higher bars indicating more activity and darker colors representing abnormal activity. Selecting a patient opened their specific clinical details, which included medications, laboratory results, vital sign measurements, and communication events, organized by hour. Trends or changes in laboratory results or medication doses were identified by placing a directional arrow adjacent to the specific item.

Acuity refers to the severity of a patient’s illness. Patients with higher acuity levels tend to be more ill and require more interventions and closer observations. Other systems make the assumption that acuity corresponds to the number of interventions. For example, nursing assignments in the hospital are determined based on the acuity of patients, and the patient-to-nurse ratio decreases among patients with higher acuity because nursing workload increases with increasing care needs. To predict the nursing workload as well as to measure a patient’s severity of illness, the Therapeutic Intervention Scoring System was developed in the 1970s.^13^ Therefore, in the first prototype, we explored the association of acuity with patient activity by presenting details on the amount of clinical activity that had taken place for a hypothetical patient during a 24-hour period (Figure 1). Activity represented the number of medications administered, laboratory tests completed, vital signs measured, and/or communication events that occurred for the hypothetical patient during each hour of the day. In this sense, activity can be used as a proxy for acuity because patients with higher acuity tend to have more activity (eg, more laboratory tests, vital sign measurements, etc). Using a familiar timeline approach, the acuity prototype organized data (including specific details on medications, laboratory tests, vital sign measurements, and communication events) by time and clinical category, providing a sense of the frequency and volume of events that took place during the 24-hour period for each hypothetical patient. The acuity prototype required 61 pages to present data for all 5 patients.

#### Prototype 2: Problem View

**Figure 2.**
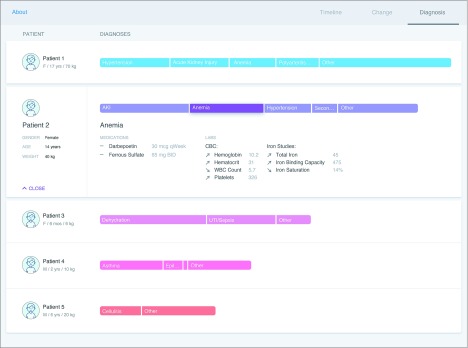
Problem Prototype The problem prototype organized data by clinical problems. Each problem was represented by a horizontal line, with its length determined by its relative priority. Tapping a problem displayed the relevant details, including pertinent medications, laboratory tests, and vital signs. Arrows placed next to results identified how details changed over time. Results, such as vital signs or communication events, that did not fit into a hypothetical patient’s clinical problems were put in the other category.

The second prototype organized data into their respective clinical problems to provide a problem-focused approach to data organization and presentation (Figure 2). Each problem was represented by a horizontal bar, with longer bars suggesting more clinical significance or priority for the hypothetical patients and problems. The length of the bar was determined by a novel algorithm we developed to assign a priority score for each problem. The algorithm took into consideration the activity level (including the number of laboratory test results, medications, vital sign data, and clinical notes) and the degree of abnormality for a specific result as well as how much a specific result changed compared with a previous value, taking into consideration the directionality of change (ie, better or worse). For example, a clinical problem associated with a greater number of medications and worsening laboratory test results would generate a higher-priority score, which we used to determine the length of the line on the display. The actual numeric scores were not displayed in the prototype. Aligning all clinical problems for a hypothetical patient on a single horizontal line provided a sense of the patient’s overall clinical priority. The problem prototype required 20 pages to present the clinical data, making it the most compact of the 3 designs.

#### Prototype 3: Change View

**Figure 3.**
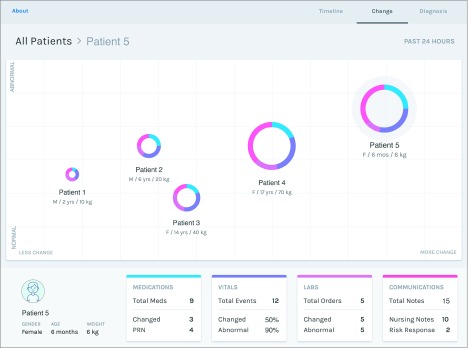
Change Prototype Hypothetical patients, represented as circles, were plotted on a grid, with the horizontal axis representing the amount of clinical change (both positive and negative) and the vertical axis representing the degree of abnormal activity. The size of each hypothetical patient’s circle was directly proportional to their activity level during the past 24 hours. Selecting an individual patient displayed patient-specific information, including details on medications, laboratory test results, vital sign measurements, and the frequency of clinical notes.

The change prototype highlighted change, abnormal activity (ie, abnormal test results or vital sign measurements, needed medication events, and risk documentation), and overall activity within a single visualization by plotting each patient on a grid (Figure 3). Patients were represented as a circle, the diameter of which was associated with the amount of patient activity (ie, larger circles indicated more activity). The location of the patient’s circle on the horizontal axis represented their degree of change, while their location on the vertical axis captured the percentage of abnormal activity during a 24-hour period. The location of patients on the grid provided a sense of their overall clinical picture. The change prototype required 38 pages to present all clinical data.

### Procedures

After obtaining consent, participants were shown a single prototype at a time, provided a brief introduction to its specific features and visualization strategies, and then asked to rank the 5 hypothetical patients in the order that they would prioritize seeing them based on each prototype’s overview (ie, initial ranking). Participants then interacted with the prototype to view each hypothetical patient’s medical record to form a more complete clinical picture for each patient. After this thorough review, participants ranked the patients again (ie, final ranking). Participants then repeated these tasks for the remaining prototypes. We compared the initial and final rankings of individual participants to evaluate the ability of each prototype’s overview visualization to accurately convey patient priority. We presented the prototypes to participants in varying order to minimize presentation bias and recorded the interaction sessions using a screen capture tool.

### Outcomes

To assess our primary outcome, we measured cognitive workload directly via the NASA Task Load Index (TLX) scale, a standardized scale that measures the subjective workload across the 6 following factors experienced by an individual when completing a specific task: mental demand, physical demand, temporal demand, performance, effort, and frustration.^14^ The NASA TLX scale has been used to assess the task-based cognitive workload experienced by health care professionals in a variety of settings, ranging from surgery^15^ to nursing^16,17^ to health care technology.^7,8,18,19^ The Agency for Healthcare Research and Quality^20^ recommends its use as a tool to assess health information technology workflows because it provides a simple method to measure a user’s cognitive workload across a variety of domains. While the NASA TLX instrument has some limitations, it has been shown to be preferred compared with other cognitive assessment tools in health care applications,^21^ with high levels of reliability,^22^ validity,^23^ and sensitivity.^24^ We used the NASA TLX to evaluate the workload associated with completing the 2 following key prioritization tasks: (1) finding information for an individual patient and (2) comparing information among patients. The NASA TLX scale generates a score from 1 to 100, with lower scores indicating reduced cognitive workload. Participants completed the NASA TLX assessment after completing each prioritization task with each prototype, resulting in 6 total scores for each participant.

After using each prototype, participants provided feedback on the usability of the prototypes to complete additional tasks performed during the clinical prioritization process via a 5-point Likert scale (ie, hard, somewhat hard, neutral, somewhat easy, easy). We asked participants to rank the prototypes in order of their personal preference at the end of the study.

### Statistical Analysis

We reported NASA TLX scores for each prototype and task as medians with interquartile ranges (IQRs) because of their nonnormal distribution. We performed linear mixed-effects regression modeling to compare the NASA TLX scores for each prototype and task. To account for participant variation in the actual NASA TLX scores, we ranked each participant’s scores for each prototype from lowest (first) to highest (third) and compared the number of rankings (ie, the number of first, second, and third ranks) for each prototype and task using χ^2^ analysis. We also looked at the use patterns associated with each prototype by comparing the mean number of pages viewed by each participant and compared these results via 1-way analysis of variance. We considered *P* ≤ .05 statistically significant, and all tests were 1-tailed. Participant preference was also compared using χ^2^ tests. All analysis was performed in R version 3.6.1 (R Project for Statistical Computing).

## Results

Invitations to 49 physicians at Seattle Children’s Hospital were sent, 32 (65%) indicated interest in participating, and 29 (59%) completed the study. Overall, 14 participants (48%) identified as women and 15 (52%) as men. The mean (range) age of our participants was 43 (35-58) years. Participants had been practicing in their respective fields for a mean (range) of 11 (3-30) years. These physicians represented a broad diversity of practices, including general and subspecialty pediatricians as well as pediatric surgeons (Table).

**Table.** Participant Characteristics

Characteristic	No. (%)
Age, mean (range), y	43 (35-58)
Men	15 (52)
Women	14 (48)
Time in practice, mean (range), y	11 (3-30)
Technology use	
Owns a smart phone	29 (100)
Time using a smart phone, mean (range), y	9 (1-15)
Owns a tablet	24 (83)
Time using a tablet, mean (range), y	5 (1-8)
Pediatric specialty	
Craniofacial	2 (7)
Critical care	2 (7)
General pediatrics or hospital medicine	10 (35)
Endocrine	1 (3)
Gastroenterology	1 (3)
Infectious disease	1 (3)
Nephrology	6 (21)
Rheumatology	2 (7)
Pulmonary	1 (3)
Surgery	3 (10)

### Assessing Cognitive Workload

For task 1, the change prototype was associated with lower median (IQR) NASA TLX scores (ie, less cognitive burden) compared with the acuity prototype (30.3 [15.2-41.6] vs 48.5 [18.7-59.3]; *P* = .02) but not compared with the problem prototype (30.3 [15.2-41.6] vs 36.6 [18.7-51.8]; *P* = .13) (eFigure 1 in the Supplement). For task 2, the change prototype was associated with lower median (IQR) NASA TLX scores compared with the problem prototype (29.1 [16.3-50.8] vs 43.5 [26.6-55.9]; *P* = .02) but not compared with the acuity prototype (29.1 [16.3-50.8] vs 39.9 [25.7-61.3]; *P* = .07) (eFigure 1 in the Supplement). In addition, the change prototype had the lowest NASA TLX scores for individual participants in 17 of 29 (59%) rankings for task 1 (χ^2^_4_ = 24.4; *P* < .001) and 18 of 29 (62%) rankings for task 2 (χ^2^_4_ = 17.2; *P* = .002).

### Secondary Outcomes

The acuity prototype had the highest agreement in patient priority ranking comparing the initial ranking with the final ranking (18 of 29 [62%]), followed by the change (12 of 29 [41%]) and problem (9 of 29 [31%]) prototypes, but the comparison was not statistically significant (χ^2^_2_ = 5.9; *P* = .054). Usability data showed that participants generally found the change prototype easier to use (eFigure 2 in the Supplement). Despite the acuity prototype having the most pages and the highest mean (SD) page views per participant (68.4 [44.2] page views) compared with the problem (56.8 [27.5] page views) and change (52.0 [29.1] page views) prototypes, this difference was not significant (*P* = .18). Significantly more participants selected the change prototype (22 of 29 [76%]) as their first choice compared with the acuity (5 of 29 [17%]) and problem (2 of 29 [7%]) prototypes (χ^2^_4_ = 36.21; *P* < .001).

## Discussion

Our results demonstrated that differences in the visualization of EHR data were associated with changes in the cognitive workload of clinical decision-making when identifying patients with high-priority care needs. The change prototype was associated with the lowest cognitive workload when completing the prioritization tasks compared with the acuity and clinical problems prototypes. Despite the similar amount of clinical information, the organization and presentation of the clinical content differed substantially among the prototypes, leading to cognitive workload differences. The lower cognitive workload associated with the use of the change design was most likely associated with the visualizations that highlighted clinically meaningful patterns. In comparison, the overview visualizations of the other prototypes did not provide enough detail or context, meaning that participants had to search for additional information to form a clinical impression, which increased their cognitive workload.

**Figure 4.**
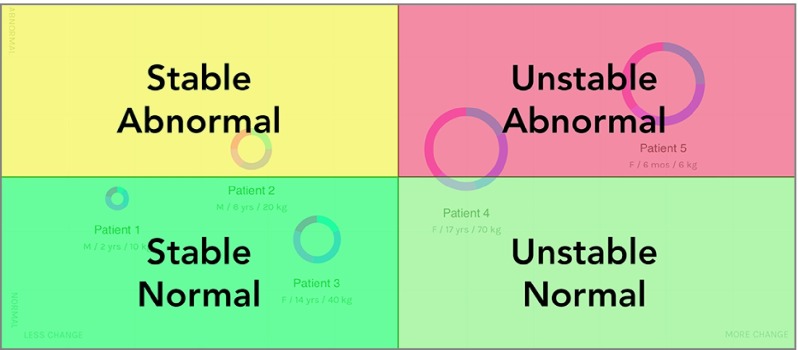
Interpretation of Change Visualization The prototype highlighting clinical change supported fast pattern recognition and decision-making by effectively organizing data into 4 quadrants. Patients found in the upper-right corner would be considered high priority because they were clinically deteriorating, while those in the lower-right corner would be considered low priority because they were clinically improving. Patients found in the upper-left corner indicate abnormal activity but with little substantial change. Finally, those in the lower-left corner indicate patients with normal activity and little change.

Traditionally, EHRs display a tremendous amount of data spread across multiple locations in the clinical record^5,25^ and therefore require physicians to search, identify, and remember important data elements used during the care process. This time-consuming and error-prone workflow continues until they have found enough details to form an impression of the patient’s status. The change prototype was associated with changes in this search-and-discovery process by highlighting and organizing key prioritization details, ie, abnormal activity and change, into a concise visualization that supported fast interpretation and projection about the clinical status of a patient or group of patients (Figure 4). For example, patients who were clinically deteriorating were clustered on the upper-right side of the grid (ie, more change and more abnormal results), while those who had clinical improvements would tend to cluster in the lower right (ie, more change and fewer abnormal results). Therefore, once orientated to the design, physicians could quickly identify patients who were likely to be the most concerning by looking in the upper-right portion of the display, while patients in the lower-right portion would be less concerning.

This organization supports preattentive processing, ie, subconsciously recognizing visual patterns, by separating items based on location, resulting in rapid pattern recognition and data interpretation. Grouping the required data used during the clinical prioritization process simplified the interpretation of clinical data because the EHR performed some of the processing that has traditionally been done by physicians, resulting in reduced cognitive workload. Importantly, the visualization did not make specific recommendations but instead provided enough details and context to allow physicians to quickly recognize and process meaningful patterns without having to search the medical record and assemble the clinical picture by memory. Given that 76% of our participants preferred the change prototype, this finding suggests that physicians are open to the EHR processing, organizing, and visualizing details that highlight clinically meaningful patterns. In addition, despite introducing a new and unfamiliar method of visualizing clinical data, the change design still had the lowest NASA TLX scores and highest usability scores of all 3 designs. This implies that novel systems should not avoid creating new kinds of visualizations if they successfully communicate clinically meaningful patterns and enhance physician decision-making.

Our work complements previous work that demonstrated that providing highly relevant details was associated with reductions in a physician’s cognitive workload when using EHRs.^7,26^ However, EHR systems have traditionally organized data into source-oriented (ie, where the data comes from, such as the laboratory, clinical notes, etc) or time-oriented (ie, data are primarily organized chronologically) views requiring physicians to search for and find the fraction of clinical details required to provide care. Concept-oriented views, in which data are organized and presented around clinical concepts, have the potential to greatly reduce the cognitive workload associated with EHR use.^27,28^ Each of our 3 prototypes was a different concept-oriented view based on the 3 patient characteristics used during the prioritization process, ie, acuity, clinical problems, and change. Given that physicians typically search for information to help them accomplish a specific clinical task (eg, prescribing medications), well-designed concept-oriented views could provide all the required data elements in a single location to support the task’s successful completion. For example, a concept-oriented medication list would provide relevant data elements associated with each medication, such as laboratory test results, physical examination findings, dosage changes, adherence data, etc, in a single location within the EHR, allowing a physician to quickly understand a patient’s response to therapy without having to search multiple EHR sections. Ahmed et al^7^ demonstrated that a novel EHR interface that reduces extraneous details and organizes details via concept-oriented views based on grouped lists of text and/or numerical data was associated with a reduction in the cognitive workload of physicians in intensive care units compared with a traditional EHR. Our work extends their findings by demonstrating that well-designed concept-oriented views that leverage information visualization, a method of communicating data visually to support fast, efficient, and accurate interpretation, were also associated with reductions in the cognitive workload.

The ability to reduce the cognitive workload through concept-oriented information visualization depends on achieving the appropriate balance between presenting clinically meaningful patterns as discrete and fully contained visualizations with the need for physicians to search, identify, and remember the details required to identify and validate the same patterns. The structure of the acuity and problem prototypes likely led to clinicians searching through additional details to provide insight into the overview visualizations, which may have been a factor in increasing the cognitive workload for our participants when compared with the change prototype. The need to spend more time searching likely explained why the acuity view had the highest concordance between the first and final ranking tasks of the 3 prototypes. With data spread across the most pages in the acuity prototype, participants likely relied on the overview visualization to form their clinical impressions because they may have found searching for the confirmatory details burdensome and overwhelming. This study provides additional evidence that designing concept-oriented visualizations requires a human-centered approach to uncover the content, organization, and visualization methods to best support the information needs of practicing physicians.^29,30^

### Limitations

This study has several limitations. First, our work focused on the cognitive workload associated with the use of 3 novel high-fidelity prototypes and did not include a production-based EHR for comparison. Although we cannot directly compare the cognitive workload of these prototypes with that of a functional EHR, our results can inform the development of future work to directly compare concept-oriented information visualization displays with existing EHR information retrieval systems. Second, the clinical data used in our 3 prototypes was synthetically created specifically for the purposes of this study. Although we used a rigorous and methodical process to create the synthetic patient data used in each prototype,^12^ unrecognized variations in complexity could exist and be associated with differences in patient data among the prototypes that influenced our results. Third, our study only included 29 physicians from a single tertiary care pediatric hospital. Future studies would benefit from a larger and more diverse group of participants. Fourth, we timed the participants completing each ranking task as an additional outcome, but because of conversations with participants during study procedures, the timings did not reflect the actual time to task completion. Without this objective measure of the time required to complete the ranking tasks, it is difficult to assess how the differences in NASA TLX scores affected our participants’ ability to prioritize their patients when using each prototype. Therefore, future assessments would benefit from including objective assessments that measure a task’s successful or efficient completion.

## Conclusions

Using well-designed, concept-oriented visualizations to highlight clinically meaningful patterns appropriately shifts the cognitive burden of information seeking from physicians to EHRs. Instead of searching, finding, and remembering the clinical details required to identify the same patterns, physicians could benefit from new EHR-derived visualizations to assist them. Freeing physicians from search-intensive tasks reduces their cognitive workload, allowing them to focus on what matters most—caring for their patients.

## Notes

Supplement. **eFigure 1. **NASA TLX Scores **eFigure 2.** Usability Scores
