# Supplementary material for: Association of Health Record Visualizations With Physicians’ Cognitive Load When Prioritizing Hospitalized Patients
Source: JAMA Netw Open. 2020 Jan 15;3(1):e1919301. doi: 10.1001/jamanetworkopen.2019.19301 (PMC6991320; doi:10.1001/jamanetworkopen.2019.19301)
Supplement: Supplement. — eFigure 1. NASA TLX Scores eFigure 2. Usability Scores [file jamanetwopen-3-e1919301-s001.pdf]

Supplementary Online Content

Pollack AH, Pratt W. Association of health record visualizations with physicians' cognitive load when prioritizing hospitalized patients. *JAMA Netw Open*. 2020;3(1):e1919301. doi:10.1001/jamanetworkopen.2019.19301

eFigure 1. NASA TLX Scores
eFigure 2. Usability Scores

This supplementary material has been provided by the authors to give readers additional information about their work.

© 2020 Pollack AH et al. *JAMA Network Open.*

eFigure 1. NASA TLX Scores

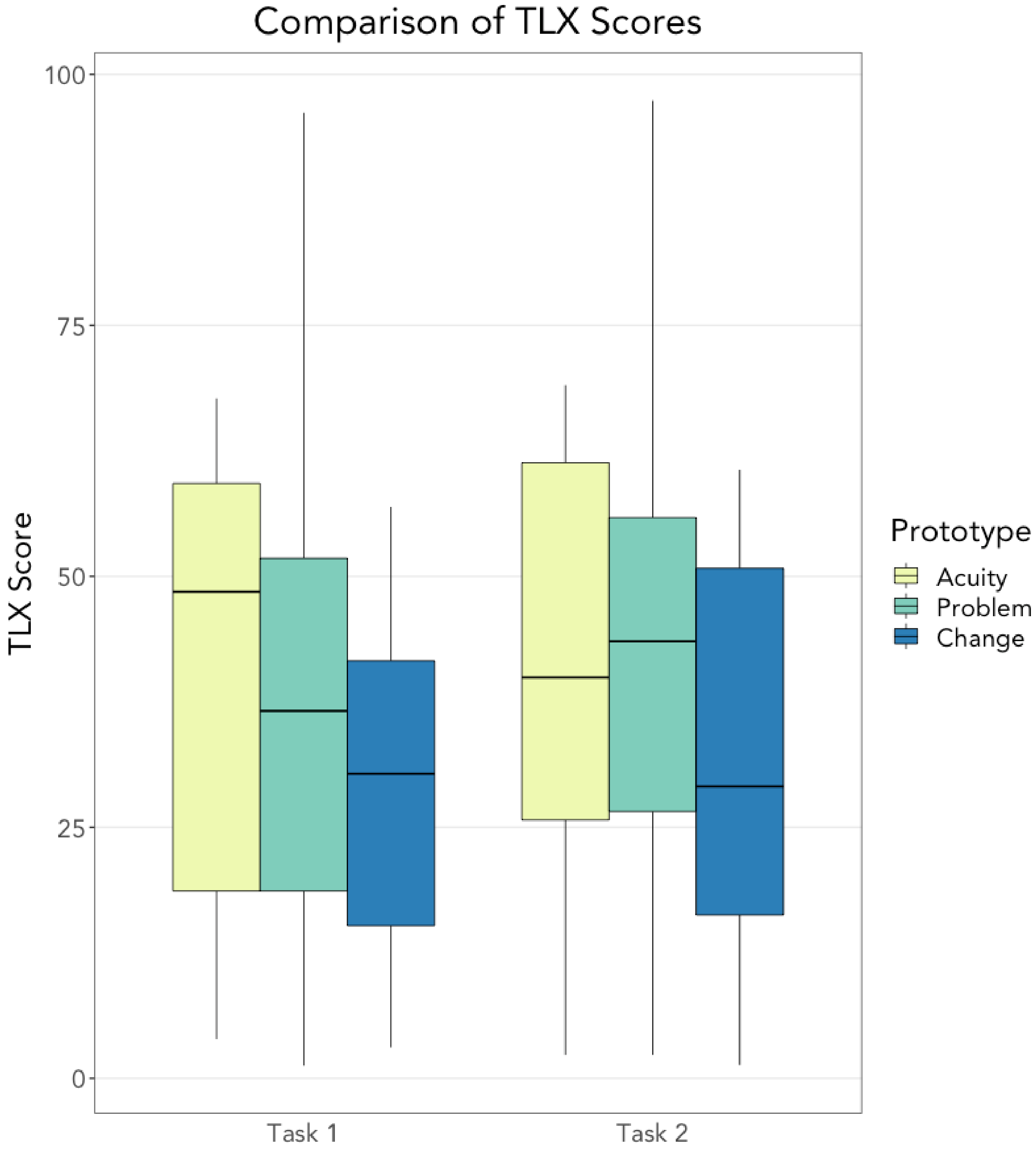

Median TLX scores and interquartile ranges for each prototype and task. Lower TLX scores indicate lower cognitive workload. For task 1 there was only a significant difference between TLX scores comparing the Change and Acuity prototypes ($p = 0.02$). For Task 2, there was only a significant difference between the Change and Problem prototypes ($p = 0.02$).

© 2020 Pollack AH et al. *JAMA Network Open.*

eFigure 2. Usability Scores

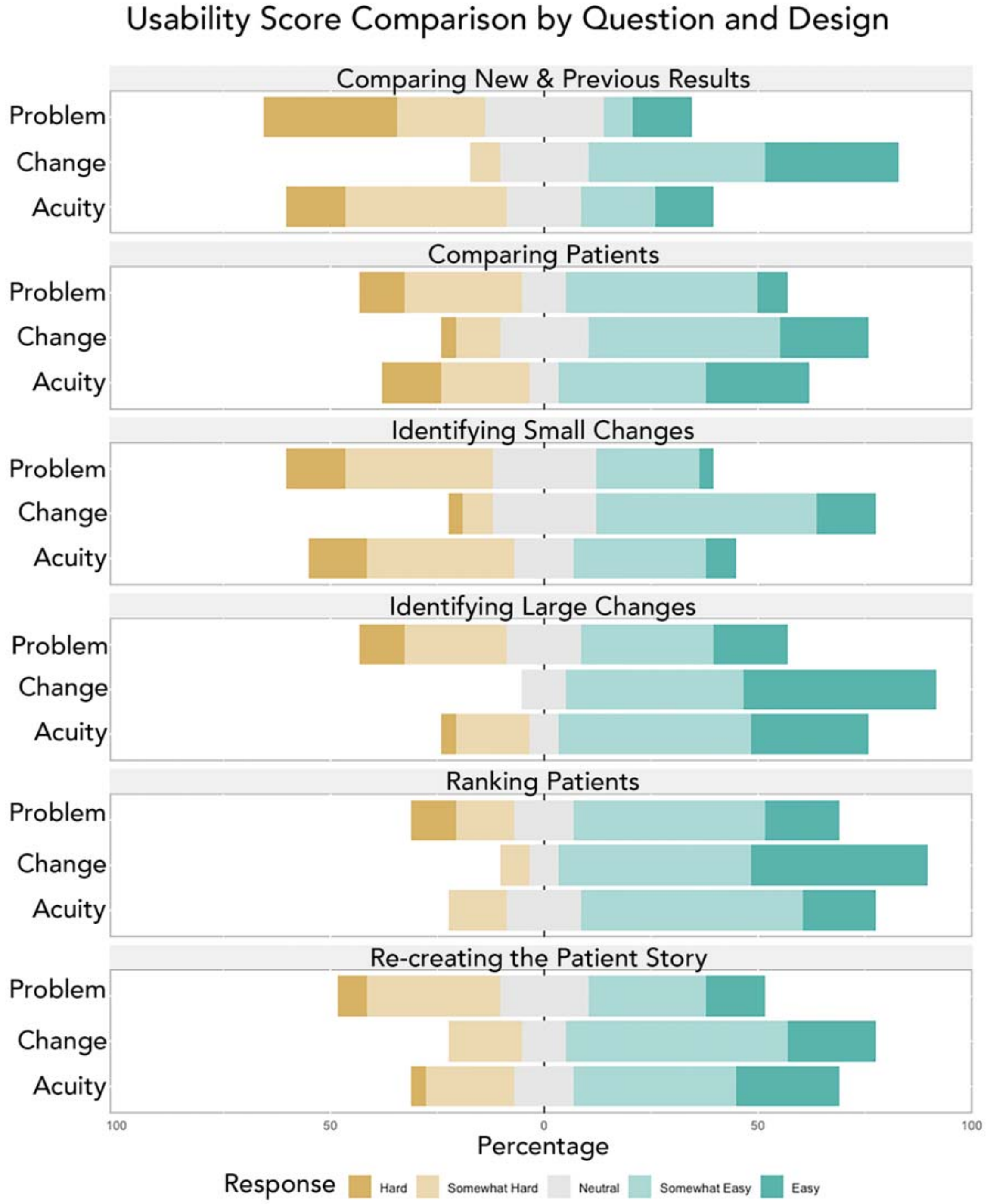

Usability data for completing 6 prioritization tasks for each of the three prototypes. Bars to the right indicate more favorable usability scores, compared to bars aligned more to the left.

© 2020 Pollack AH et al. *JAMA Network Open.*
